# Influence of the Tensile Strain on Electron Transport of Ultra-Thin SiC Nanowires

**DOI:** 10.3390/molecules29030723

**Published:** 2024-02-04

**Authors:** Qin Tan, Jie Li, Kun Liu, Rukai Liu, Vladimir Skuratov

**Affiliations:** 1 School of Materials Science and Engineering, Jiangsu University of Science and Technology, Zhenjiang 212100, China; t_t5980xmttkx@163.com (Q.T.); liurukai085@163.com (R.L.); 2 Joint Institute for Nuclear Research, Dubna 141980, Russia; skuratov@jinr.ru

**Keywords:** electronic properties, tensile strain, SiC NWs, ultra-thin, simulations and calculations

## Abstract

The influence of nanomechanical tensile behavior on electron transport is especially interesting for ultra-thin SiC nanowires (NWs) with different diameters. Our studies theoretically show that these NWs can hold stable electron transmission in some strain ranges and that stretching can enhance the electron transmission around the Fermi level (E_F_) at the strains over 0.5 without fracture for a single-atom SiC chain and at the strains not over 0.5 for thicker SiC NWs. For each size of SiC NW, the tensile strain has a tiny effect on the number of device density of states (DDOSs) peaks but can increase the values. Freshly broken SiC NWs also show certain values of DDOSs around E_F_. The maximum DDOS increases significantly with the diameter, but interestingly, the DDOS at E_F_ shows little difference among the three sizes of devices in the late stage of the stretching. Essentially, high electron transmission is influenced by high DDOSs and delocalized electronic states. Analysis of electron localization functions (ELFs) indicates that appropriate tensile stress can promote continuous electronic distributions to contribute electron transport, while excessively large stretching deformation of SiC NWs would split electronic distributions and consequently hinder the movement of electrons. These results provide strong theoretical support for the use of ultra-thin SiC NWs in nano-sensors for functional and controllable electronic devices.

## 1. Introduction

Semiconductor nanowires (NWs) are essential in the construction of nanoelectronic and nanophotonic devices, and they have shown significant promise for integrated nanosystems [1,2,3]. Notably, among the most promising nanoscale building blocks that can function in challenging conditions and at high temperatures, high powers, and high frequencies are silicon carbide (SiC) NWs [4,5,6,7], since they incorporate quantum-size effects [8,9] with the exceptional qualities of SiC, such as high electron mobility, strong breakdown electronic field, high thermal conductance, good mechanical properties, good chemical stability, good field-emitting performance, radiation resistance, and others [10,11,12,13,14]. The majority of NWs are of a cubic zinc-blend structure (-SiC) orientated along the (111) direction, with mechanical characteristics superior to other forms of SiC [15,16].

Understanding the mechanical properties of SiC NWs under external loads is critical to their practical application as electronic or optical interconnects and microelectromechanical components. Experimentally, Zhang et al.’s report indicated that β-SiC nanowires up to 150 nm in diameter have a very unique mechanical response and provided direct evidence of nanoscale super-plasticity and the fracture processes of a single SiC nanowire at low temperatures [17]. Han et al., also proved that ceramic SiC NWs, about 80 nm in diameter, have very large strain plasticity at temperatures close to room temperature [18]. β-SiC NW linear elastic deformation was found until brittle fracture based on quantitative mechanical characterization of SiC NWs from 45 nm to 17 nm by an in situ tensile test [19]. Theoretically, the nanomechanical behaviors of (111)-oriented SiC NWs depending on the diameter were studied by Makeev et al. using molecular dynamics (MD) simulations with a diameter of [1 nm, 4 nm] [20]. The diameter of Wang et al.’s study was only 2 nm but considered multiple lengths and temperatures [21]. The SiC NWs studied by Tsuzuki et al. and Wang et al. were about 2 nm to 5 nm. Tsuzuki et al. focused on the stretching behaviors of SiC NWs ranging from 2 nm to 5 nm in diameter but at several high and extremely high strain rates [22]. In contrast, Wang et al. revealed the effect of stacking defects, point defects, twins, intergranular amorphous film, and surface morphology on the brittleness and plasticity of (111)-oriented SiC NWs [23].

The diameter of β-SiC NWs has shown a decreasing trend in the study of nano-tensile behaviors, and as the diameter decreased, the SiC NWs have shown better mechanical response and greater strain plasticity. Therefore, it is important to study SiC NWs with a smaller diameter. However, for ultra-thin SiC NWs with less than 0.5 nm in diameter, there are not enough published studies on their tensile behavior. Furthermore, taking into account both the constraints of experimental settings and cost, as well as established simulation methods, pertinent atomistic simulation and theoretical calculation studies are important and necessary for studying the nanomechanical behaviors of SiC NWs with small diameters.

In related research on the nanomechanical behaviors of SiC NWs, the influence of nanomechanical behaviors on electrical properties is a highly attractive topic due to its great practical value and theoretical importance. SiC NWs have been described as having a wide range of uses in the electronics industry, particularly in harsh conditions. When working, SiC NWs, especially thin NWs, are likely to stretch unintentionally, which can unavoidably affect the electrical characteristics and the device’s overall performance [9,24]. Therefore, understanding the relationship between the mechanical stress and the electronic characteristics or electron transport mechanism can help avoid catastrophic failure when working in harsh environments and is key to the practical applications of SiC NWs-based devices in a mechanically disturbed environment, especially for ultra-thin SiC NWs. Furthermore, based on such relationships, high-performance stress/pressure sensors can be made with ultra-thin SiC NWs [7] and applying mechanical stress can be used as one effective way to control electrical properties by means of the tensile behavior [25,26]. SiC NWs could be a promising material for electrical control switches. 

Gao et al., have conducted a relevant experimental study which demonstrated a decrease in the resistance with increasing compressed strains for 6H-SiC NWs with a diameter of 170 nm, regarded as the piezoresistance effect. Such effect has been attributed to the strain-induced changes in surface states of SiC NWs. Stable and repeatable I–V curves through multiple-voltage sweeping have been achieved by C-AFM [27]. Shao et al. have performed in situ tensile processes on (111) SiC NWs with diameter of 156 nm and measured the corresponding I-V relationship with a transmission electron microscope (TEM) to study the piezoresistive. It has been found that the conductivity increases monotonically as the tensile strain increases, and the current growth rate remains constant until the break [28]. With the new fixing method, in situ TEM electromechanical coupling measurements have been performed by Cui et al. on pristine 3C-SiC NWs with a diameter of 249 nm. The study has revealed that the piezoresistance coefficients are 2 to 4 times smaller than those of their corresponding bulk counterparts [29]. Recently, Wu et al. have reported a strategy for enhancing electrical response influenced by different external compressive forces by growing a ZnO nanolayer around the nanowire surface by ALD [30]. Also, Yan et al. have theoretically studied the influence of axis stress on electronic properties, especially the bang-gap of (111)-oriented SiC NWs, and the diameter is from 0.61 nm to 1.23 nm [31]. Recently, Oliveira et al. have explained the relationship between the strain and the band structures of (111)-oriented SiC NWs with diameters of 0.71 nm to 1.42 nm [32]. 

Clearly, the diameter of SiC NWs in relevant experimental studies is relatively large, emphasizing the measurable electrical properties and corresponding measurements. Related theoretical studies have only highlighted the effect of the strain on energy band structures of SiC NWs. The diameter ranges from about 0.6 nm to 1.4 nm, which is even less than the previous study of tensile behaviors. However, for ultra-thin SiC NWs with diameters less than 0.5 nm, the impact of the stress on electronic properties, especially the electron transport mechanisms or internal device electronic structures during a tensile process, has not yet been revealed. Due to the excellent performance of SiC NWs in previous studies and their high research value, the electron transport behaviors of ultra-thin SiC NWs during their tensile process are highly anticipated.

## 2. Results and Discussion

**Figure 1:**
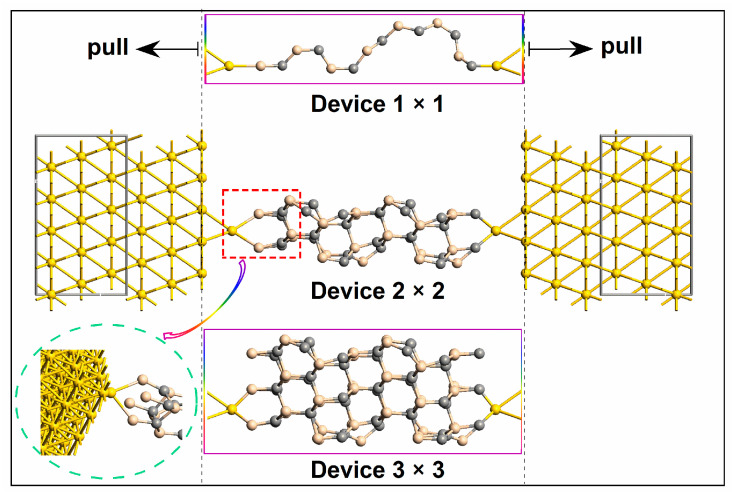
Schematic diagram of SiC NW initial devices with three cross-sectional areas. The inset shows the detailed connection between the electrode and SiC NW of Device 2 × 2.

We employ Virtual NanoLab to construct electronic devices with more than 15 Å of vacuum space in a supercell, which enables electrostatic interactions to decompose within the system. Figure 1 illustrates the structures of the electronic devices that contain three sizes of ultra-thin SiC nanowires sandwiched between two 5 × 5 Au (111) electrodes. These three types of SiC NWs are along the crystal direction (111) with cross-sectional areas of one atom, four atoms, and nine atoms, respectively. They have the same length. These three types of SiC NWs are called Wire 1 ×, Wire 2 × 2, and Wire 3 × 3, and the corresponding devices are labeled as Device 1 × 1, Device 2 × 2, and Device 3 × 3, in a similar manner. To maintain chemical and geometrical stability between the SiC NW and each electrode, one Au atom is connected to each side of each SiC NW as a linked atom. For Wire 1 × 1, the Si atom is the bonding agent for the electrode. For thicker SiC NWs, the electrode is attached to the central Si atoms in a hollow manner. For example, the inset gives the detailed connection mode between the electrode and SiC NW of Device 2 × 2. The mechanical stretch is along the length direction, displayed in Figure 1.

To investigate the influence of strain on the electron transport properties of ultra-thin SiC NW-based electronic devices, we selected representative strains and their associated structures, according to the relationship between the strain and stress. Seven typical strains are selected for both Wire 1 × 1 and Wire 2 × 2, and thirteen for Wire 3 × 3. Detailed information is shown in Table 1. For clarity, we divide the tensile process of each nanowire into three tensile stages (initial stage, middle stage, and late stage), as detailed in Table 1 below, based on the number of representative strains selected. The strain represents the relative change in length and is the ratio of the shape variable to the original length dimension, which has no unit. Here, we express the strain in decimals.

### 2.1. Equilibrium Electron Transmission

**Figure 2:**
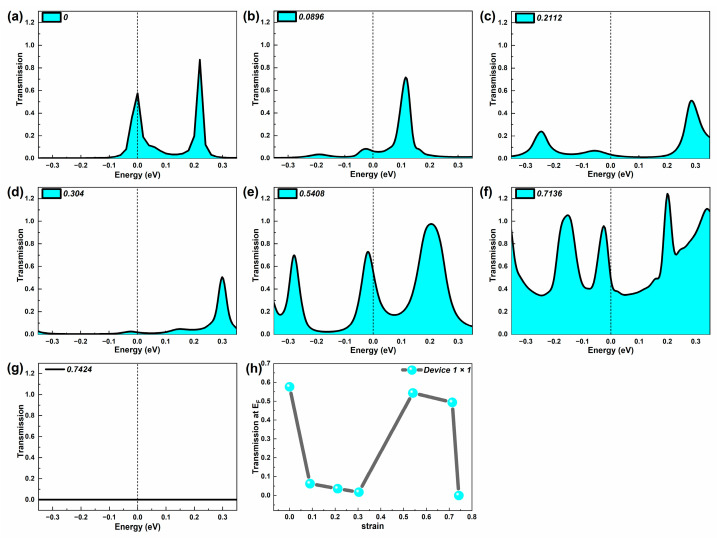
(**a**–**g**) Equilibrium electron transmission spectrum of Device 1 × 1 around the Fermi level at representative strains, respectively. (**h**) Equilibrium electron transmission coefficient of Device 1 × 1 at the Fermi level under typical strains.

**Figure 3:**
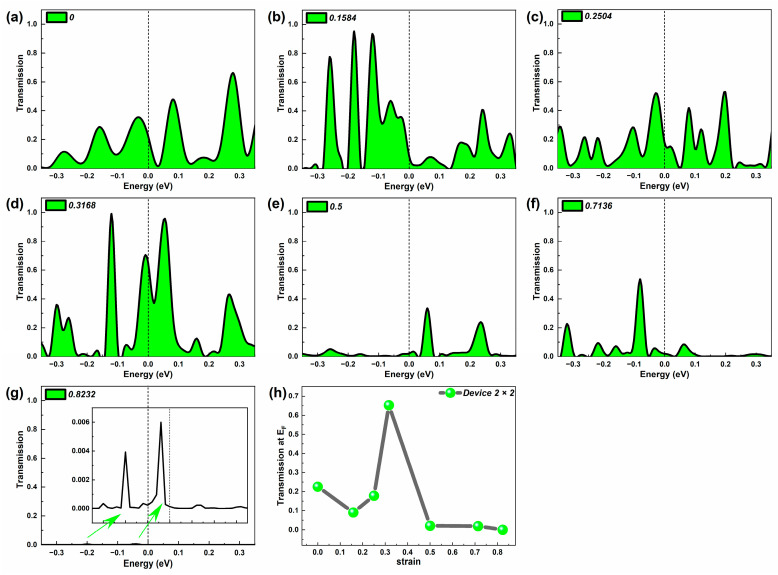
(**a**–**g**) Equilibrium electron transmission spectrum of Device 2 × 2 around the Fermi level at representative strains, respectively; the inset shows the transmission spectrum in a narrower possibility range. (**h**) Equilibrium electron transmission coefficient of Device 2 × 2 at the Fermi level under typical strains.

**Figure 4:**
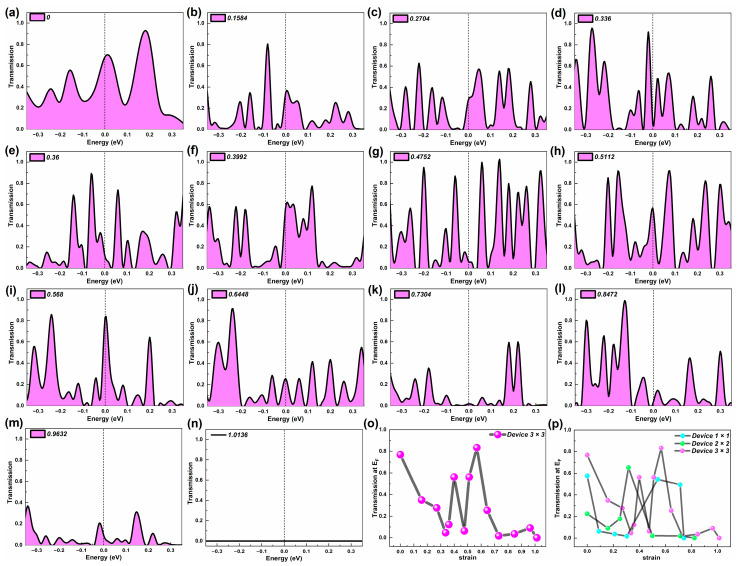
(**a–n**) Equilibrium electron transmission spectrum of Device 3 × 3 around the Fermi level at representative strains, respectively. (**o**) Equilibrium electron transmission coefficient of Device 3 × 3 at the Fermi level under typical strains. (p) Comparison of equilibrium electron transmission coefficient at the Fermi level under typical strains among Device 1 × 1, Device 2 × 2 and Device 3 × 3.

Firstly, we calculated the equilibrium electron transmission spectrum of three devices at representative strains which can reflect their electron transport capacity, shown in Figure 2, Figure 3 and Figure 4. Furthermore, the equilibrium electron transmission at the Fermi level (E_F_) under representative strains is also given in these figures. By diagonalizing the transmission matrix, we can obtain the transmission eigenvalues, and then by summing the transmission eigenvalues of all the electron transmission orbitals at E_F_, we can obtain the electron transmission at E_F_ [33]. When the device is at zero bias, the electron transmission around E_F_, especially at E_F_, is higher for devices with strong electron transmission capacity [34]. For the electron transmission spectrum of the probe device, the number, width, position, and peak value of the transmission peaks are all factors that cannot be ignored.

Figure 2 shows the electron transmission spectrum of Device 1 × 1 around E_F_ under various representative strains. For the number of transmission peaks, as the tensile process begins, the number of transmission peaks of Device 1 × 1 appears to change firstly from a small amount (initial-stage) to multi-volume (middle and late stages), and finally to 0. Next is the width of the transmission peak. Similar to the change in the number of peaks, the change in transmission peak width with the tensile process shows a shift from narrower peaks (initial-stage) to wider peaks (middle and late stages), and finally the transmission peaks no longer appear. This is followed by a discussion of peak values, and in order to provide a more comprehensive analysis of the peak changes, we select the closest transmission peaks to the Fermi energy level (including transmission peaks at E_F_) named Category I peaks. The peaks with the highest peaks in the entire [−3.5 eV, 3.5 eV] energy range are named Category II peaks. When Wire 1 × 1 is unstretched, the summit of Category I peak is at E_F_. At the beginning of the stretching, the Category I peak gradually moves away from E_F_ and the values of the Category I and II peaks decrease. The transmission efficient at E_F_ also falls off rapidly. When the strain reaches 0.5408 or over but does not fracture, the Category I peak is very close to E_F_ and the Category II peak elevates greatly. The number of high transmission peaks surges. When Wire 1 × 1 breaks, no transmission can be observed. 

Based on this discussion, it can be found that the trend of the peak value of the first type of peak with the strain is similar to the trend of the electron transmission coefficient at E_F_ (shown in Figure 2h). But the maximum peak of the first type of peak occurs late at the strain of 0.7136, which is different from the E_F_, where the maximum electron transmission coefficient appears in the initial state (strain 0). The changing trend of the second type of peak is similar to that of the first category, with the largest peaks occurring late at the strain of 0.7136. In all, the maximum peaks of both the first and second peaks occur late in the stretch (strain 0.7136). From Figure 2h, the good transmission possibility at E_F_ clearly appears in the un-tensile wire and the stretching wire at the middle and late stages.

Figure 3 shows an electron transmission spectrum of Device 2 × 2 around E_F_ under various representative strains. Similar to Device 1 × 1, we analyze the number of peaks, the width of the peaks, and the peak value. Device 2 × 2 obviously shows many more transmission peaks than Device 1 × 1 does, whatever the strain is. As the SiC nanowires are stretched, the number of transmission peaks increases initially. When the strain reaches 0.5, the number of peaks drops obviously until Wire 2 × 2 breaks. For the transmission peak width, the transmission peak width of Device 2 × 2 remains basically the same throughout the rest of the stretch, except for the larger peak width in the initial state and the narrow peak width at the end of the stretch. The peak analysis, similar to the Device 1 analysis, is discussed in the Category I peak and Category II peak. When the strain does not exceed 0.5, the Category I peak is very close to E_F_, with an increased peak value, and the summit of the Category II peak fluctuates. When the strain reaches to 0.5, the Category I peak stays a little farther away from E_F_ but with a much lower peak value, and the summit of the Category II peak drops fast. As the strain continue to rise without fracture, the peak value of Category II peak increases again.

Since the first type of peak is close to E_F_, the trend of its peak value with tensile change is similar to the trend of the electron projection coefficient at E_F_ with tensile change in Figure 3h. But for the second type of peak, the change in the peak value with the strain is very different from the trend of the first type of peak. Although the second type of maximum peak still occurs in the middle of the stretch, there is also a larger Type II peak in the initial stage, At the end of the stretch, the peak of the second category also rises before falling. It differs from the Class Platform Phase presented late in the stretch at the first peaks and at E_F_. From Figure 3h, the strongest electron transmission at E_F_ appears in the middle stage (at strain of 0.3168). When the strain reaches 0.5 and above, Device 2 × 2 presents weaker electron transport. Thus, by applying the appropriate stress, the electron transmission of Device 2 × 2 can be improved significantly. A large degree of stretching would suppress the electron transport for Wire 2 × 2.

Figure 4 gives the electron transmission spectrum of Device 3 × 3 around E_F_ under various representative strains. In quantity, the number of transmission peaks in Device 3 × 3 is significantly greater than in the first two, and as the stretch progresses, the number of peaks is presented by a small number in the initial stage, multi-vector in the middle stage, and a gradual transition from multiple to zero in the late stage. In addition to the larger transmission peak width in the initial stage, the width of the transmission peak during the rest of the tensile process is basically the same. The Category I peak stays very close to E_F_, influenced by the tensile strain. Split peak occurs closest to E_F_ in the initial and middle stages. When the strain exceeds 0.568, most of the transmission peaks present obviously lower peak transmission, especially Category I peak. Furthermore, the peak value of the Category II peak is very high under most of the strains, except when it is about to break. 

Similar to Device 2 × 2, the changing trend of the peak value of the first class is similar to that of the transmission electron coefficient at E_F_ (shown in Figure 3e). For the peaks of the second category, the maximum peaks of the second category of Device 3 × 3 occur in the middle of the stretch (at strain 0.4752). In addition, large peaks and those numerically close to the maximum peaks of the second category occur repeatedly in the initial state (strain to 0) and middle of the stretch. At the late stage of tensile (strain 0.8472), there is also a large peak value of the second type, which is very different from the trend of the electron transmission coefficient at E_F_ changing with the tensile strain.

Finally, a longitudinal comparison is made of the cross-sectional atomic area of nanowires as an argument. As the cross-sectional area of SiC NWs becomes larger, the number of peaks gradually increases, the average width of peaks gradually increases, and the maximum electron transmission coefficient at E_F_ gradually increases. From Figure 3e, the electron transmission coefficient at E_F_ waves in the initial and middle stretching stages, where stronger electron transmission appears. Device 3 × 3 presents the great electron transport at the strain of 0.568. In the late stage, the electron transport stays weak.

Therefore, for SiC nanowires with cross-sectional areas that are not single atoms (i.e., Device 2 × 2 and Device 3 × 3), the ultra-thin SiC NWs that remain in the initial state always show fewer transmission peaks and wider transmission peaks, compared with the NWs that undergo tensile strain, at the scales of their respective size. This shows that for SiC nanowires with large cross-sectional areas, a certain stretching strain can make the electronic transition of the device easy. As a result, the electronic transport performance of the device can be improved to some extent. 

From the relationship between the equilibrium electron transmission and strain at E_F_, it is not hard to see that the SiC nanowires of various sizes have certain similarities caused by the destruction of their original structure. The reduction in the equilibrium electron transmission of the nanowires is affected by tensile strain. However, when the strain increases, the electron transmission of the ultra-thin 3C-SiC NWs can be somewhat adjusted due to some specific structures. This suggests that tensile strain can also affect the equilibrium electron transmission of these NWs.

### 2.2. Electronic Structures

**Figure 5:**
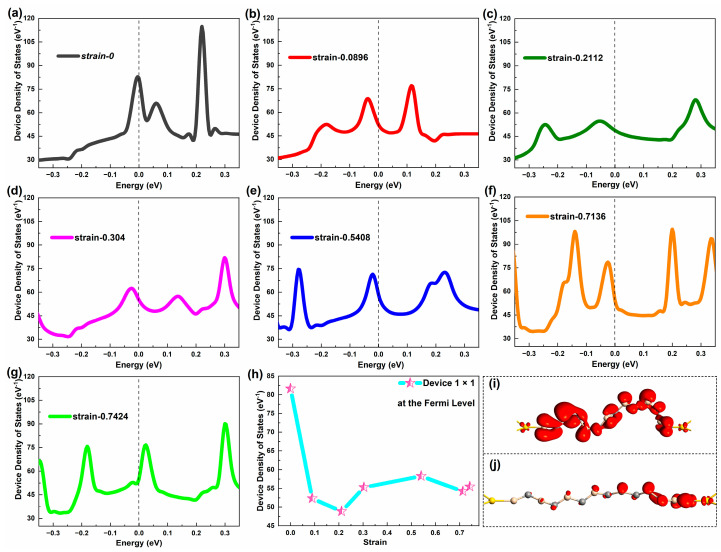
(**a**–**g**) Equilibrium device density of states of Device 1 × 1 around the Fermi level at representative strains, respectively. (**h**) Equilibrium device density of states of Device 1 × 1 at the Fermi level under typical strains. (**i–j**) Equilibrium electronic states of Device 1 × 1 at strain 0.0896 and 0.2112, respectively.

The device density of states (DDOSs) represents the electronic structure of the device [35]. In general, the higher the state density value around E_F_, the more electrons around E_F_. To some extent, there will be more electrons crossing the energy gap. However, the actual situation remains constrained by other factors. Figure 5a–g exhibits the DDOS of Device 1 × 1 around E_F_. In the [−3.5 eV, 3.5 eV] range of energy levels, the number of DDOSs peaks throughout the stretch of Device 1 × 1 remains from three to four peaks. When not stretched, a significant DDOS peak is exactly at E_F_. As the stretch progresses, the peak gradually deviates from E_F_, and the peak decreases. In the middle of the stretch, the peak of DDOS becomes not very obvious. However, after the stretch, the peak becomes apparent again, but the DDOS peak closest to E_F_ is not yet at E_F_. It is visible that for Device 1 × 1, the DDOS values of the stretched devices are lower than the unstretched device. Different from the electron transmission, the DDOS of Device 1 × 1 still shows certain values around E_F_ when it is broken. However, the tensile strain will cause a large deformation of the entire nanowire throughout the tensile process, but it has little effect on the electronic structure of the device.

To give a quantitative relation more clearly, Figure 5h presents the changing trend of the DDOS value at E_F_ with strain. It can be found that when Device 1 × 1 is not stretched, the DDOS value at E_F_. is largest. The DDOS value at E_F_ is minimized in the beginning of the stretch. The corresponding DDOS value is minimum at strain 0.2112. As the stretch progresses, the DDOS value picks up slightly in the middle and late stages of the stretch and stabilizes, but the DDOS value is much smaller than that before the stretch. The stretching would reduce the DDOS at E_F_ of Device 1 × 1. Comparing Figure 2 with Figure 5, the DDOS curves are similar with the transmission curves in shape under the influence of the strain. Moreover, the prominent DDOS peaks basically correspond to the transmission peaks. By comparing Figure 2h with Figure 5h, for Device 1 × 1, the trend of the electron transmission coefficient changing with strain in the initial stage is consistent with that of the DDOS values with strain. The structure of SiC NWs with a cross-sectional area of one atom is relatively simple, so the DDOS can fully reflect the electronic structure of Device 1 × 1, which also directly determines the electronic transport performance of Device 1 × 1.

Based on this, Figure 5(i-j) gives the distribution of the electron states at the two maxima of DDOSs. It can be found that when the DDOS value is greatest, the electronic state of the device is distributed uniformly along the nanowires and is in an extended state. When the DDOS value is minimal, the electronic state of the device is localized, and the nanowire near the leakage electrode is fuller than the source electrode. The delocalization of electronic states results in an increase in the SiC NWs’ charge transfer ability, which indicates that the coupling of the SiC NWs and electrodes has a small barrier that enhances the device’s electron transmission [36]. This also shows that the extended electronic states distribution makes the DDOS value of nanowires higher, while the distribution of the local electronic states makes the DDOS value smaller. When a SiC NW is a single atomic chain, the tensile strain applied to both ends of the nanowire changes the electronic state distribution and the electronic structure of the device.

**Figure 6:**
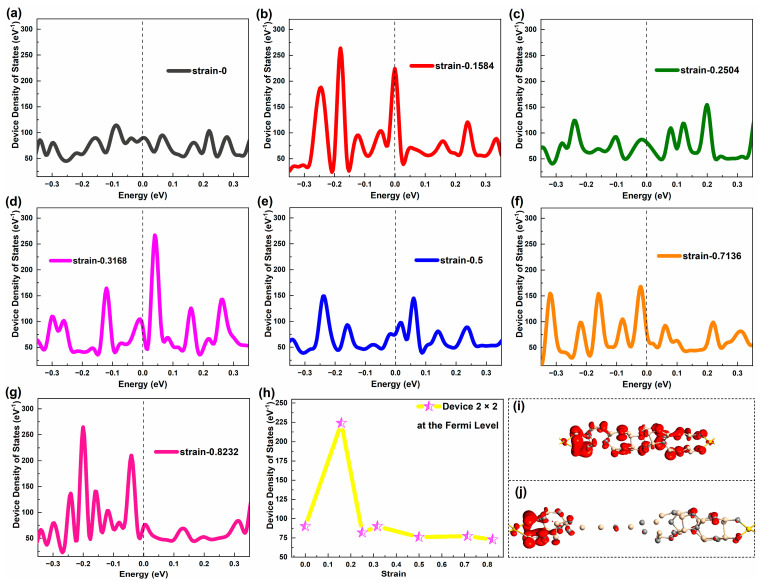
(**a**–**g**) Equilibrium device density of states of Device 2 × 2 around the Fermi level at representative strains, respectively. (**h**) Equilibrium device density of states of Device 2 × 2 at the Fermi level under typical strains. (**i–j**) Equilibrium electronic states of Device 2 × 2 at strain 0.1584 and 0.8112, respectively.

Figure 6a–g shows a DDOSs map of Device 2 × 2 around E_F_ under representative strain. In terms of the number of peaks, Device 2 × 2 generally has more state density peaks than Device 1 × 1 in the same energy range, and the maximum peak is also larger than Device 1 × 1. This shows that the electronic structure of the SiC NWs with a cross-sectional area of four atoms is more complex than that of the SiC NW with single atomic chains. Unlike Device 1 × 1, Device 2 × 2 presents a gentle distribution of DDOSs near E_F_ when not stretched. When the tensile stress is applied, the DDOSs peak increases significantly. Especially, in the early stretch period, the DDOSs peak increases significantly and is just at E_F_. As the stretch progresses, the peak decreases and deviates from E_F_. But the peaks are much higher than when not stretched. At the end of the stretch, when the nanowire is just pulled off, the obvious DDOS peak is mainly distributed on the left side of E_F_. Affected by the tensile stress, Device 2 × 2 shows not many obvious DDOS peaks around E_F_, only presenting one or two prominent DDOS peaks at strains of 0.1584 and 0.3168. It should be noted that different from the electron transmission spectrum, the DDOS of Device 2 × 2 still shows certain values around E_F_ when it is broken.

Comparing Figure 3 with Figure 6, the DDOS curves of Device 2 × 2 are similar with the transmission curves in shape under the influence of the strain and the prominent DDOSs peaks basically correspond to transmission peaks, which is similar with Device 1 × 1. Figure 6h shows how the DDOS values of Device 2 × 2 at E_F_ vary with strain. It can be found that the maximum DDOS value of Device 2 × 2 occurs during the entire stretch (strain 0.1584), with the lowest value in the late stretch (strain 0.8232). At other strains, the DDOS values of Device 2 × 2 at E_F_ are not much different from when not stretched. 

Combined with the distribution of electronic states (see Figure 6(i-j) for analysis, it can be found that the electronic states of Device 2 × 2 present a uniform distribution over the nanowires at the strain of 0.1584. In the case of strain 0.8112, the electronic states of the device are quite localized, are mainly concentrated near the leakage electrode of the device, and there is almost no electron state distribution near the source electrode. This also shows that the DDOS value at E_F_ is closely related to the electronic states near E_F_. In addition, the maximum DDOS value of the device occurs in the pre-stretching phase and the value of the maximum DDOS differs greatly from the initial state. For SiC NWs with a cross-sectional area of four atoms, slight tensile strain can make the electronic states distribution and electronic structure of the device more complex. To some extent, it may be helpful to improve the electronic transport capability of devices.

**Figure 7:**
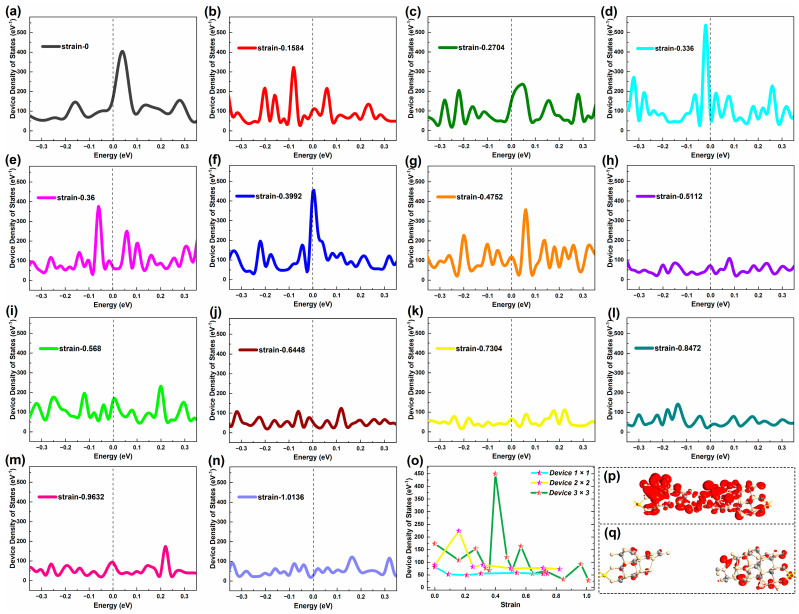
(**a–n**) Equilibrium device density of states of Device 3 × 3 around the Fermi level at representative strains, respectively. (**o**) Comparison of equilibrium device density of states at the Fermi level under typical strains among Device 1 × 1, Device 2 × 2 and Device 3 × 3, respectively. (**p–q**) Equilibrium electronic states of Device 3 × 3 at strain 0.4752 and 1.0136, respectively.

Figure 7 gives the DDOSs map of Device 3 × 3 near E_F_ under various representative strains. Not surprisingly, SiC nanowire devices with a cross-sectional area of nine atoms have more peaks than the previous two. And the peak has grown to more than 500 eV^−1^. This shows that the electronic structure of the device is more complex than in the first two cases. When not stretched, the only peaks in the entire interval are more pronounced and near E_F_. At the beginning of tensile period, the number of DDOS peaks increases, especially the obvious peaks, but the peaks are not high compared with the unstretched one. As the stretch progresses, the number of peaks changes little, but gradually highlights the single peak. However, when the strain exceeds 0.5, until it breaks, the summit of the DDOSs peak decreases and a gentle trend occurs. And the DDOS hardly changes in the middle and late stretch, which shows gentle and similar distributions starting from the strain of 0.5112, characterized by low multi-peaks. Comparing Figure 4 with Figure 7, the DDOS peaks approximate the transmission peaks in quantity but differ in the height of peak for Device 3 × 3. Similar to other sizes of devices, the freshly broken Device 3 × 3 also show certain values of DDOSs around E_F_.

Figure 7o gives the DDOS values of the three devices at E_F_ under each typical strain. Obviously, when the strain is 0.3992, Device 3 × 3 has the largest DDOS value, while DDOS is the smallest when the strain is 1.0136. During other strains, the DDOS varies little. On this basis, we combine the distribution of electronic states (Figure 7(p-q)) to analyze. It can be found that the electronic states of Device 3 × 3 are uniformly distributed at the maximum DDOS value, whether near the source electrode, near the leakage electrode, or in the middle of the nanowire. Inversely, when the DDOS value is minimal, small and discrete electronic states are distributed locally around the source and leakage electrodes due to the tensile fracture of the nanowires. This phenomenon proves again that the DDOS value at E_F_ of the device can reflect the electronic states and the electronic structure of the device near E_F_.

In addition, the maximum DDOS value of Device 3 × 3 appears in the middle of the stretch and the minimum DDOS value appears later in the stretch. Both the maximum and minimum values differ significantly from the DDOS values near E_F_ in the initial state of Device 3 × 3. This shows that for SiC NWs with a cross-sectional area of nine atoms, a certain amount of tensile strain can effectively increase the DDOS value of the device and improve the electronic distribution and electronic structure of devices. But excessive tensile strain can also have counterproductive effects and makes the electronic states distribution of the device discrete.

For Device 2 × 2 and Device 3 × 3, the trend of DDOS changing with strain during the pre-stretch and late-stretch periods is not fully matched with the trend of electron transmission with strain, based on the contrast of Figure 3h and Figure 6h, as well as Figure 4o and Figure 7o. This is due to the large cross-sectional area of the SiC NWs, unlike the simple Device 1 × 1. The devices with larger cross-sectional SiC NWs contain more Si atoms and C atoms, which have more influence on each other, so the DDOS values do not appear to be more consistent with the electron transmission than Device 1 × 1.

We can see that as the cross-sectional area becomes larger, the number of peaks in the state density map of the SiC NWs increases and the peaks increase. This shows that the cross-sectional area of the SiC NWs gradually increases from one atom to nine atoms, thus increasing the influence between the Si atoms and the C atoms in the devices, and the overall electronic structure of the devices becomes more complex. From the contrast of DDOS at E_F_ among the three devices (Figure 7o), Device 1 × 1 presents lower DDOS values than Device 2 × 2 under various strains. Device 1 × 1 in an unstretched state shows very similar DDOS values with Device 2 × 2 at most strains, except the strain of 0.1584 with the maximum. As the diameter increases, the DDOS fluctuates with the strain, not showing the changing trend as well as the devices with thinner SiC NWs. Device 3 × 3 displays several local optimal points, which are higher than the DDOS values of the devices with thinner SiC NWs at all strains except the maximum of Device 2 × 2. Noticeably, the maximum DDOS of Device 3 × 3 is significantly greater than other devices, which is about two times the maximum DDOS of Device 2 × 2 and more than six times the DDOS of thinner devices at most strains. 

It is worth noting that the devices with large cross-sectional areas or smaller cross-sectional areas tend to have similar trend of DDOS values with strain to electron transmission coefficients with strain in the late stage of the stretch. This is because at this point the nanowire stretch basically ends and the complete structure of the NW has been destroyed. This reduces the interaction between atoms and atoms and the factors that constrain electrons from crossing the energy gap. The electronic states near E_F_ determine the electronic transport capability of the device again, so the trend of the DDOS value at E_F_ with strain is similar to that of the electron transmission coefficient with strain.

**Figure 8:**
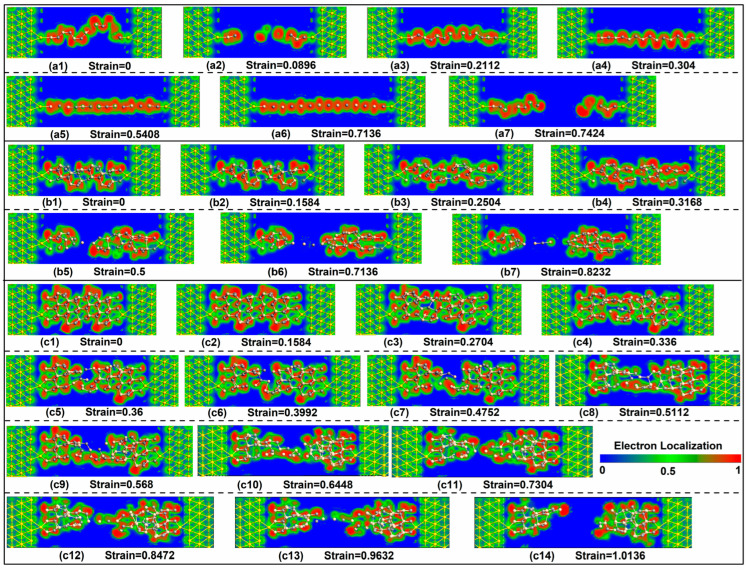
(**a1**–**c14**) ELF maps of the devices with Wire 1 × 1, Wire 2 × 2, and Wire 3 × 3 at representative strains, respectively.

In order to investigate thoroughly the internal mechanism of electron transport properties, we studied the electron distributions of the devices with these stretched SiC NWs by calculating their electron localization functions (ELFs), which are given in Figure 8. The ELFs can be described in the form of a contour plot in real space with values ranging from 0 to 1, and a large value denotes a high degree of electron localization. Generally, values of 1 or 0.5 represent the fully localized or fully delocalized electrons, respectively, while a value near 0 refers to a very low electron density. The colors of red and blue refer to the highest (1) and lowest (0) values of ELF, indicating accumulation and depletion of electrons at different colored regions, respectively.

From Figure 8, it can be seen that the electrode extension part of each device almost has the same distribution of electrons. On the whole, the red color can be found in the surroundings of many Si and C atoms for each SiC NW, revealing that the electrons around these atoms possess stubborn localization, denoting strong covalent electron states which can electronically stabilize the frame of SiC NWs. More interestingly, a green color can be seen around many red areas, demonstrating that the homogeneous electron gas is widely distributed in outskirts where these electrons are free and can transfer flexibly. Such distribution of electronic states would provide bases for electron transport. A blue color can be observed in the areas far away from the SiC NWs and the parts with larger tensile deformations (the ELF value is 0), which testifies to the electron deficiency.

In detail, for Wire 1 × 1, the unstretched state possesses the continuous green area from the left to the right that forms the whole electron transfer path, together with the shortest length and less tortuous degree, contributing stronger electron transmission than other stretched states. Wire 1 × 1 at strains of 0.5408 and 0.7136 shows the most stretchable and straightest green strips (distribution of electronic states), which facilitates the electron transferring. So, the two tensile states also display stronger electron transmission. When Wire 1 × 1 breaks, the electron distribution area appears to be divided into two parts with a continuous blue area, leading to weaker electron transport. As the diameter increases, several small blue patches also appear in the electronic distribution inside the SiC NWs. For Wire 2 × 2, the stretched state at the strain of 0.3168 presents the least small blue patches and the biggest green areas in the electronic distribution inside the SiC NWs, bringing about the strongest electron transport. Larger strains initiate continuous blue areas inside the NWs and smaller strains cause more small blue patches and less green areas in electronic distribution inside the SiC NWs, which are not conducive to the electron transport. Similarly, the electronic distributions inside the SiC NWs are also important for Wire 3 × 3.

## 3. Computational Methods

To conduct simulated tensile tests on ultra-thin SiC NWs, we utilize the LAMMPS software package that is large-scale and massively parallel [37], and we perform these tests based on molecular dynamics (MD) simulations [38]. P-p-p boundary conditions are used to construct the NWs. One layer of atoms is secured on both sides and NWs are stretched in the direction of z (see Figure 1). The strain rate is determined by the complete evolution of NW structures, with a level of 0.0008 ps^−1^. The Tersoff potential function has been opted for. The timestep is 0.001 ps. It should be noted that previous work has shown that the empirical calculating methods may produce anomalous stress−strain curves, especially qualitatively incorrect maximum strength/stain [39]. Here, we use ordinary semi-local functionals to predict strains, so the maximum strain values may not be very accurate. However, our research is mainly on the changes in electron transport properties during the whole stretching process, not targeted to the specific values of extreme strains. Still, more accurate strains can be obtained by the dispersion corrections with adequate dispersion coefficients [39].

Using the first-principles theory (density functional theory combined with non-equilibrium Green’s functional theory), we calculate the electron transport properties by Atomistix Toolkit (ATK) package [40]. To enhance accuracy, we choose local atomic numerical orbitals that have double-zeta polarized basis sets, and MGGA is selected as the exchange correlation with a fitting parameter c (1.108) [41]. K-point sampling is set to 3 × 3 × 50. The density mesh cut-off for the electrostatics potential is 75 Ha. The electron temperature is set as 300 K. 

The quantum mechanical transmission probability of electrons can be given as [41].

(1) $$T\left(E,V\right)=tr\left[\Gamma_{L}\left(E,V\right)G^{R}\left(E,V\right)\Gamma_{R}\left(E,V\right)G^{A}\left(E,V\right)\right]$$

where *G^R^* and *G^A^* are the retarded and advanced Green functions of the conductor part, respectively, and ΓL and ΓR are the coupling functions to the left and right electrodes, respectively.

## 4. Conclusions

Here, we have studied theoretically the influence of the tensile strain on electron transport properties for ultra-thin SiC-(111) NWs with different sizes. The mechanical stretching behavior has been found to have a significant impact on the equilibrium electron transport, as suggested by research. For the single-atom SiC chain, slight strain would significantly reduce electron transmission, but the similarly strong electron transmission appears again in the late stretching stage without fracture, while for thicker SiC NWs, stronger electron transmission occurs in the middle stretching stage. The largest electron transmission coefficient, which appears during stretching, gets better as the diameter of the SiC NW increases. Interestingly, the influence of strain on electronic structures differs among these three sizes of SiC NWs. The overall distribution of DDOS around E_F_ is not affected largely by the tensile strain for the single-atom SiC chain, while the DDOS at E_F_ reduces significantly once the strain is applied but varies smoothly during stretching. For the thicker SiC NW, several obvious DDOS peaks would form under the influence of strain, compared with the flat distribution of the unstretched one. The largest DDOS appears in the initial stretching stage and larger strains make the DDOS as low as the unstretched state. For the thickest one, little strain causes a minor alteration in the general distribution of DDOS around E_F_, and it would keep flat when the strain exceeds 0.5. As the diameter grows, the strain at which the largest DDOS value at E_F_ emerges enhances, which is in the middle stretching stage. Furthermore, a large device density of states and delocalized electronic states contribute to strong electron transmission. Proper tensile stress can cause continuous electronic distributions to contribute to electron transport, while excessively larger stretching deformation of SiC NWs would split electronic distributions and consequently hinder the movement of electrons. Strong theoretical backing is provided by these results for the use of ultra-thin SiC NWs in functional/controllable electronic devices, as well as nano-sensors.

## Figures and Tables

**Table 1:** Typical strains selected for three sizes of ultra-thin SiC NWs.

Wire	Stress–strain	Typical Strains	Tensile Stage
1 × 1	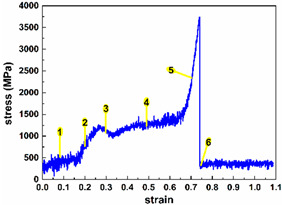	0.0896, 0.2112	Initial stage
		0.304, 0.5408	Middle stage
		0.7136, 0.7424	Late stage
2 × 2	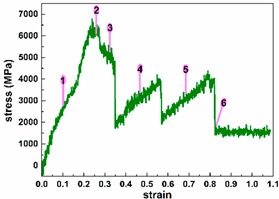	0.1584, 0.2504	Initial stage
		0.3168, 0.5	Middle stage
		0.7136, 0.8232	Late stage
3 × 3	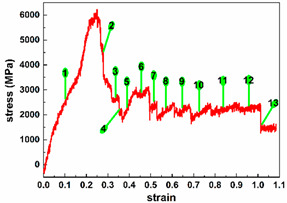	0.1584, 0.2704, 0.336, 0.36, 0.3992	Initial stage
		0.4752, 0.5112, 0.568, 0.6448, 0.7304	Middle stage
		0.8472, 0.9632, 1.0136	Late stage

## Data Availability

Data will be made available on request.
